# Reproductive efficiency of sows inseminated at single dose fixed time with refrigerated, cryopreserved and encapsulated spermatozoa

**DOI:** 10.1111/rda.14179

**Published:** 2022-06-20

**Authors:** Raúl Sánchez‐Sánchez, Sonia S. Pérez‐Garnelo, Mercedes Martín Lluch, Paloma de la Cruz, Maria V. Falceto, Alejandro Córdova‐Izquierdo, Juan Grandía Torner, Olga Mitjana, Andrés E. Suárez, Teresa Montull, Juan Grandía Ansó, Trinidad Ansó, Ernesto Gómez‐Fidalgo

**Affiliations:** ^1^ Department of Animal Reproduction National Institute for Agricultural and Food Research and Technology (INIA‐CSIC) Madrid Spain; ^2^ Department of Animal Pathology Veterinary Faculty of Zaragoza Zaragoza Spain; ^3^ Autonomous Metropolitan University Xochimilco Unit México City Mexico; ^4^ Agrotest Control Zaragoza Spain; ^5^ Porcine Artificial Insemination Center of Cinco Villas, Ejea de los Caballeros Zaragoza Spain

**Keywords:** Buserelin, cryopreserved semen, encapsulated spermatozoa, fixed‐time insemination, refrigerated semen, semen preservation

## Abstract

The aim was to assess the reproductive efficiency of different techniques used to preserve spermatozoa in artificial insemination semen doses (AI‐doses) by evaluating refrigeration at 15°C, cryopreservation and encapsulation. Forty‐two hyperprolific sows were treated with buserelin and inseminated once at a single fixed time. The fertility rate, embryonic vesicles viability and the early embryonic mortality (arrested conceptuses) evaluated post‐mortem at 24th day of pregnancy, were analysed in order to assess the effectiveness of each proposed technique. Results show an overall reduction on fertility using the three proposal sperm preservation techniques (69.27%, 60.00% and 78.75% for refrigerated, frozen–thawed and encapsulated AI‐doses, respectively). Total number of embryonic vesicles was very similar among the three treatments; yet, the number of viable vesicles was numerically different among groups, and thus, embryonic viability was 79.25%, 80.0% and 87.15% for refrigerated, frozen–thawed and encapsulated AI‐doses, respectively.

## 1 INTRODUCTION

Artificial insemination (AI) has changed significantly in the pig industry over the last two decades due to the major advances in the processes involved in the production of artificial insemination semen doses (AI‐doses) and its application. Refrigeration at 15°C is the most widely used technique for sperm preservation in swine, which allows semen storage for 1 to 7 days depending on extender used, intrinsic semen quality of the boar and handling laboratory conditions of semen samples. Yet, the use of frozen–thawed AI‐doses is less frequent (Pezo et al., 2019) and limited to genetic improvement or genetic resources conservation programmes.

At present, one of the most interesting strategies for pig farms is to reduce the number of AI‐doses applied per oestrus and sow, and thus, the current trend is to use a single insemination dose (De Rensis & Kirkwood, 2016). For this, a possible alternative is to control ovulation time, either by using GnRH agonists (Knox et al., 2014) or by monitoring ovarian follicular dynamics with ultrasonography (Williams & Luzbel de la Sota, 2017). Another approach is the use of encapsulated spermatozoa allowing sperm gradual release within the female reproductive tract (Faustini et al., 2012; Vigo et al., 2009), which can prolong sperm viability in the reproductive tract of sows and also the fertilization at ovulation time.

The aim of the study was to assess the reproductive efficacy of three semen preservation techniques using a single seminal dose and performing AI at a fixed time, after the use of an agonist of GnRH for oestrous and ovulation synchronization. Reproductive performance was evaluated at 24th day of gestation (D24) on hyperprolific sows, which allowed us to evaluate fertility and embryo viability in the early stages of pregnancy.

## 2 MATERIALS AND METHODS

### 2.1 Animals

The study was carried out in a commercial farm on a total of 42 hyperprolific DanBred sows, distributed in the three experimental groups as shown in Table 1. Animals were managed, cared and slaughtered during the study according to the European Community Standards (Council Directive 2008/120/EC).

**TABLE 1:** Fertility and embryo viability and embryo mortality (non‐viable vesicles with arrested conceptuses) at 24th day of pregnancy with refrigerated, encapsulated and frozen–thawed AI‐doses and a single fixed time AI (mean ± SEM)

Treatment	Number of sows	Fertility (%)	Embryonic vesicles				
			Total number	Viable		Non‐viable/arrested	
				Mean number	%	Mean number	%
Refrigerated	13	69.23 ± 13.12	21.56 ± 2.56	17.11 ± 2.01	79.25 ± 3.55	4.44 ± 0.88	20.75 ± 4.11
Encapsulated	14	78.75 ± 12.64	21.88 ± 2.71	18.88 ± 2.13	87.15 ± 3.76	3.00 ± 0.70	12.84 ± 2.84
Frozen–thawed	15	60.00 ± 12.21	21.57 ± 2.90	17.00 ± 2.27	80.00 ± 4.02	4.57 ± 1.13	20.00 ± 4.12

### 2.2 Seminal doses (AI‐doses) preparation

Semen was obtained from two boars from the ‘Cinco Villas’ AI Center, and pool of ejaculates from these boars were used for preparing AI‐doses for post‐cervical AI. The AI‐doses of the three experimental groups (frozen, refrigerated and encapsulated) were prepared in a volume of 50 ml with a total sperm concentration of 1.5 × 10^9^ sperm/dose. The methodology used in each group was as follows:

- *Frozen–thawed AI‐doses* Ejaculates were collected, evaluated for quality and frozen 30 days before AIs. After collection, samples were diluted (1:3; v:v) in the extender and transported to the laboratory (Department of Animal Reproduction, INIA; Madrid) for further cryopreservation using the extenders and freezing protocol described by Gil et al. (1996). Frozen samples were transported to the farm in a cryogenic storage tank and thawed in a water bath before AIs.
- *Refrigerated semen* AI‐doses were prepared at the AI Center (Cinco Villas) the day of AIs as usually are prepared for routine distribution to commercial farms. After collection, ejaculates from the two boars were pooled, and the resulting sample was split a half for preparing refrigerated and encapsulated AI‐doses.
  - *Liquid refrigerated AI‐doses* After semen quality evaluation, samples were diluted in Duragen® extender (Magapor) and placed in a portable cooler at 15°C until use in AI.
  - *Encapsulated AI‐doses* After semen quality evaluation, pooled sperm samples were encapsulated following the methodology previously described by Vigo et al. (2009) with some modifications. Briefly, after gelification of semen samples the solution was extruded through a peristaltic pump into a Ca^++^‐enriched solution that allowed the rapid formation of the capsules. Final size of the formed capsules was 1.5 mm in diameter. As above, semen samples were stored at 15°C until use.
  Semen motility in the three experimental groups was subjectively evaluated at the AI Center prior its transport to the farm. The minimum motility required for AI was 80% and 60% for refrigerated (liquid and encapsulated) and frozen–thawed AI‐doses, respectively.

### 2.3 Oestrous synchronization and artificial insemination

At 85 h after weaning, sows received 10 μg of intramuscular buserelin (Porceptal®, MSD), and treated sows were inseminated once 30 h after buserelin injection using a single fixed time AI with a post‐cervical catheter.

### 2.4 Embryonic development at slaughter

**FIGURE 1:**
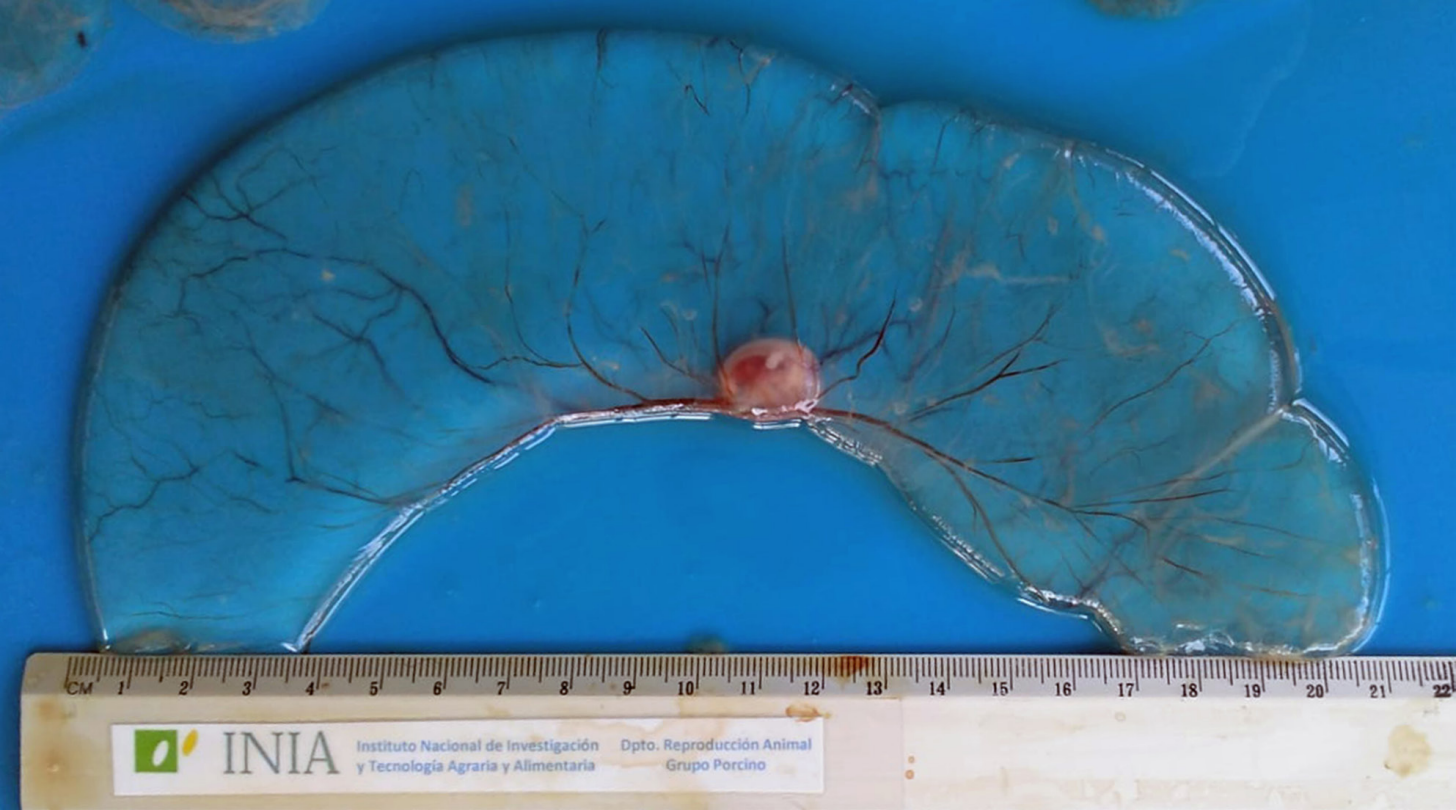
Viable embryonic vesicle at D24 post‐AI

**FIGURE 2:**
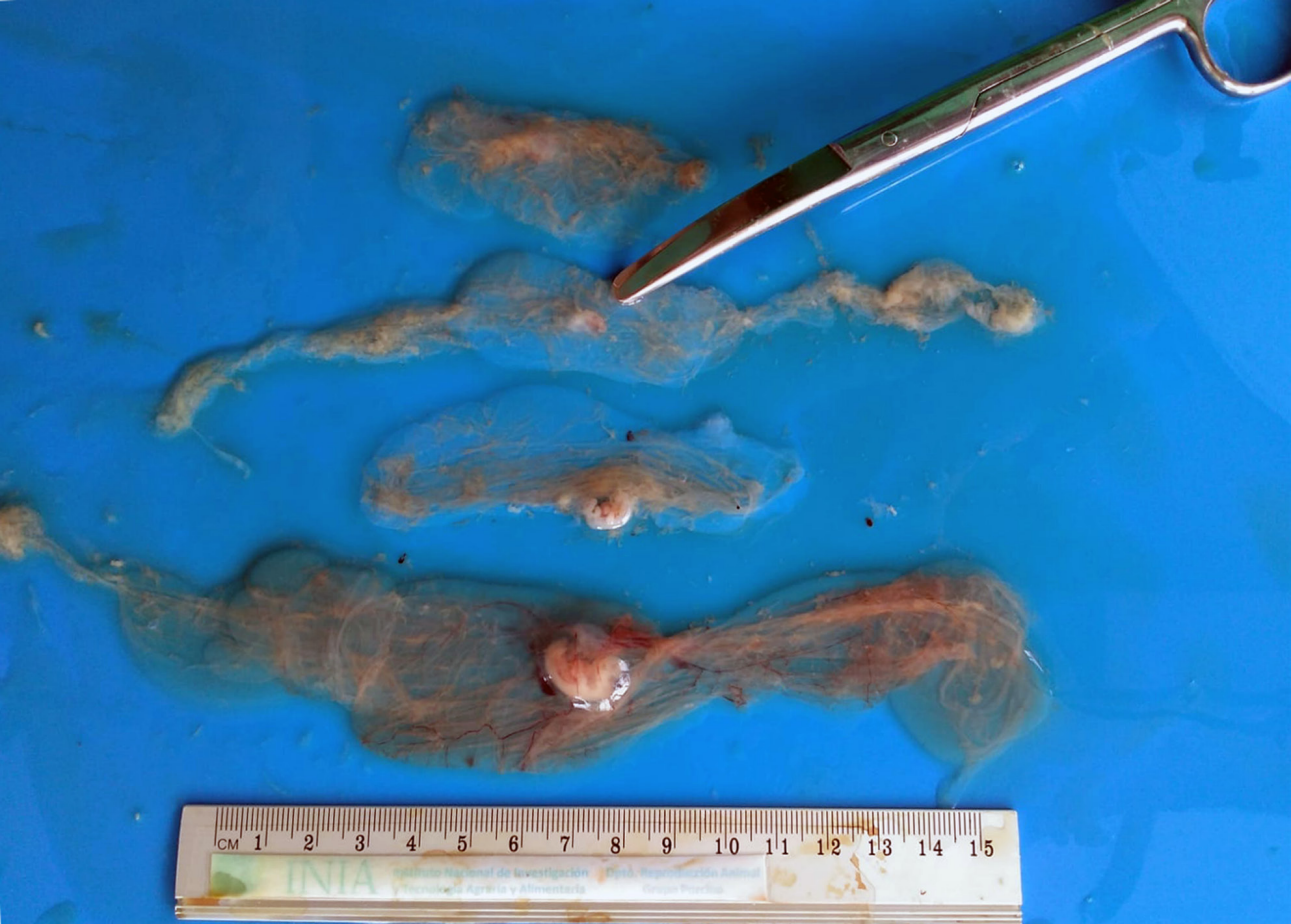
Non‐viable embryonic vesicles (arrested conceptuses) at D24 post‐AI

Sows were slaughtered on day 24 after AI. Genital tracts were transported to the Veterinary Faculty of the ‘Universidad de Zaragoza’ for further processing. After washing, uterine horns were detached from the mesometrium and longitudinally extended in order to make a longitudinal incision along the entire length of each uterine horn, and the number and viability of embryonic vesicles were assessed according to Martinez et al. (2020). Only vesicles showing a clear chorion blood supply, transparent allanto‐amniotic fluid and embryos with clearly defined tissues were considered viable (Figures 1 and 2). Sows were considered unfertilized when no embryonic vesicles were found in the uterine horns, so the fertility rate was calculated as the number of females with embryonic vesicles in the uterine horns (D24), over the total inseminated. Post‐implantation (D24) embryo viability and mortality were assessed taking into account the number of viable or non‐viable vesicles (arrested conceptuses) over the total number counted, respectively.

### 2.5 Statistical analysis

Statistical analysis was performed using SAS® 9.1 (SAS Inst. Inc.). The effect of semen treatment on fertility, total number of embryonic vesicles, total viable and non‐viable vesicles, percentage of viable and non‐viable (embryo mortality) vesicles at D24 was analysed using Least Square Means (SAS MIXED procedure). Data are presented as Mean ± Standard Error of the Mean (SEM). Statistical significance was set at *p* < .05.

## 3 RESULTS

No statistical differences were found on analysed parameters among the three methodologies used for AI‐doses preparation (Table 1), although differences on fertility were of 9.52% between refrigerated and encapsulated AI‐doses (69.23 ± 13.12 vs. 78.75 ± 12.64%; *p* > .05) and 18.75% higher with encapsulated AI‐doses than with frozen–thawed ones (78.75 ± 12.64 vs. 60.00 ± 12.21%; *p* > .05). Percentage of embryonic viability was also numerically higher with encapsulated AI‐doses than with refrigerated (7.90%) or frozen–thawed AI‐doses (7.15%), but differences were not significant (*p* > .05; Table 1).

## 4 DISCUSSION

The use of refrigerated AI‐doses at 15°C is the most widely used system for semen preservation in AI swine programmes due to its high efficacy, ease of application in farms, low cost and wide scope to use (from 1 to 7 days). Yet, semen cryopreservation is not a common semen preservation system in pig AI Centers, since reproductive results are lower and show a great variability among sires due to inter‐individual differences in semen freezability. Regarding encapsulated spermatozoa, not many results are published at farm level, but our research group maintains a line of studies to evaluate the efficacy of this technique under field conditions using different AI protocols (single insemination and traditional vs. post‐cervical insemination), and our results have been similar to those obtained with traditional methods and comparable with those published in previous studies using encapsulated AI‐doses (Vigo et al., 2009).

The fertility rate achieved in this study is low compared with the results obtained in production farms, which can exceed 90% fertility rate using routine AI techniques, with the application of at least two AI‐doses per oestrus. Yet, when a single fixed time AI is used after the application of an ovulation synchronization treatment (using with a GnRH agonist), results are controversial. Thus, similar results have been previously reported with respect routine AI techniques (Driancourt et al., 2013), but also lower (Knox et al., 2014), as in our case. The best fertility results were obtained with encapsulated spermatozoa (78.75%) compared with refrigerated (69.23%) and frozen–thawed semen in which a marked decrease in fertility (60.00%) was observed, but without reaching statistical significance. Finally, fertility results obtained with the application of frozen–thawed AI‐doses were similar to those obtained in previous studies (Yeste et al., 2017).

Total number of embryonic vesicles in uterine horns was similar among treatments (from 21.5 to 21.8). Yet, viable embryonic vesicles were numerically higher with the use of encapsulated AI‐doses (18.88) compared with the found when using refrigerated AI‐doses (17.11) or frozen–thawed (17.00), but differences did not reach statistical significance.

The low fertility results obtained with the three treatments could be explained by an inadequate response to ovulation synchronization treatment with the use of a single AI, since the pooled AI‐doses used were prepared from two proven fertility studs, and the study was carried out in multiparous females. Yet, this did not seem to affect the number of embryonic vesicles obtained at D24, since the results correspond to those described for hyperprolific sows. It is important to highlight the higher fertility and embryo viability results obtained when encapsulated AI‐doses were used when compared with the other two treatments, although the differences were not significant. Frozen–thawed AI‐doses provided low fertility, but an acceptable embryo viability that could have been reached by the use of excellent quality ejaculates or by its special resistance to the freezing–thawing protocol (Gil et al., 1996), which would also support the inadequate response of females to ovulation synchronization.

In conclusion, with the methodologies that have been used, fertility was reduced regardless of the semen preservation method used, although the reduction was more pronounced with the use of frozen–thawed AI‐doses than with the other two treatments. Embryo viability at 24 days of gestation varies according to the AI‐doses preservation methodology, obtaining better results with encapsulated AI‐doses, since with this treatment a greater number of viable embryonic vesicles were present.

## AUTHOR CONTRIBUTIONS

R.S.S. contributed to conceptualization; R.S.S., S.S.P.G., M.M.L., P.C.V., E.G.F., M.V.F., J.G.T., O.M., A.E.S., T.A., T.M. and J.G.A. contributed to methodology; R.S.S. and T.A. contributed to resources; A.C.I., R.S.S., S.S.P.G. and E.G.F. contributed to review; R.S.S. and E.G.F contributed to writing—original draft; S.S.P.G. contributed to writing—review and editing.

## CONFLICT OF INTEREST

The authors declare that they have no competing interests.

## Data Availability

Research data are not shared.
